# Supplementary material for: Tricalcium Phosphate as a Bone Substitute to Treat Massive Acetabular Bone Defects in Hip Revision Surgery: A Systematic Review and Initial Clinical Experience with 11 Cases
Source: J Clin Med. 2023 Feb 24;12(5):1820. doi: 10.3390/jcm12051820 (PMC10003370; doi:10.3390/jcm12051820)
Supplement: Supplementary file 1 [file jcm-12-01820-s001.zip › jcm-2134767-supplementary.pdf]

Table S1. Adverse events in the included studies.

Authors<br>year	Surgery management not involving the acetabular cup	Conservative management
Haenle M. et al.<br>2013	2 open reductions for dislocations<br>1 revision of head and liner<br>1 resuturing of gluteus medius for gluteal insufficiency	No
Whitehouse M.R. et al.<br>2013	2 internal fixations for femoral periprosthetic fractures<br>2 strut graftings of the femur<br>1 stem revision for aseptic loosening<br>1 stem revision for fracture<br>1 liner revision and abductor repair for recurrent dislocation<br>1 liner revision<br>1 radical debridement for infection	1 close reduction for dislocation
Whitehouse M.R. et al.<br>2013	2 debridement for early infection<br>2 strut graftings of the femur<br>1 stem revision for aseptic loosening	1 close reduction for dislocation<br>1 examination under anesthetic to assess subluxation<br>1 stem revision for aseptic loosening
Schwartz C. et al.<br>2015	No	Crutch or walking stick remained necessary in 5 of the cases
Hayashi S. et al.<br>2017	NR	NR
Abdelazim H. et al.<br>2020	1 debridement and exchange of the modular prosthesis head for early infection	5 persistent pain revolved after a mean of 4m
Gagala J. et al.<br>2021	1 internal fixation for femoral periprosthesis fracture<br>1 reoperation for metal cable broken for the osteotomy	1 close reduction for early dislocation
Comba L.C. et al.<br>2022	No	1 early infection treat with antibiotics

m, months; NR, not reported.
